# Evaluation of the Efficacy of a Bacteriophage in the Treatment of Pneumonia Induced by Multidrug Resistance *Klebsiella pneumoniae* in Mice

**DOI:** 10.1155/2015/752930

**Published:** 2015-03-23

**Authors:** Fang Cao, Xitao Wang, Linhui Wang, Zhen Li, Jian Che, Lili Wang, Xiaoyu Li, Zhenhui Cao, Jiancheng Zhang, Liji Jin, Yongping Xu

**Affiliations:** ^1^School of Life Science and Biotechnology, Dalian University of Technology, Dalian 116024, China; ^2^Orthopedics Research Center, Affiliated Zhongshan Hospital of Dalian University, Dalian 116001, China; ^3^Ministry of Education Center for Food Safety of Animal Origin, Dalian University of Technology, Dalian 116620, China

## Abstract

Multidrug-resistant* Klebsiella pneumoniae* (MRKP) has steadily grown beyond antibiotic control. However, a bacteriophage is considered to be a potential antibiotic alternative for treating bacterial infections. In this study, a lytic bacteriophage, phage 1513, was isolated using a clinical MRKP isolate KP 1513 as the host and was characterized. It produced a clear plaque with a halo and was classified as Siphoviridae. It had a short latent period of 30 min, a burst size of 264 and could inhibit KP 1513 growth *in vitro* with a dose-dependent pattern. Intranasal administration of a single dose of 2 × 10^9^ PFU/mouse 2 h after KP 1513 inoculation was able to protect mice against lethal pneumonia. In a sublethal pneumonia model, phage-treated mice exhibited a lower level of* K. pneumoniae* burden in the lungs as compared to the untreated control. These mice lost less body weight and exhibited lower levels of inflammatory cytokines in their lungs. Lung lesion conditions were obviously improved by phage therapy. Therefore, phage 1513 has a great effect *in vitro* and *in vivo*, which has potential to be used as an alternative to an antibiotic treatment of pneumonia that is caused by the multidrug-resistant* K. pneumoniae*.

## 1. Introduction

*Klebsiella pneumoniae* (*K. pneumoniae*) is one of the most common gram-negative bacteria that is responsible for hospital-acquired bacterial infections, including pneumonia, bacteremia, urinary tract infections, liver abscess, and wound infections in immune compromised patients [1, 2]. Pneumonia that is caused by* K. pneumoniae* is usually associated with a high mortality rate. In fact, despite the development of broad-spectrum antibiotics, mortality rates greater than 50% have been reported in those infected with* Klebsiella* pneumonia [3]. In addition, treating* Klebsiella pneumoniae* has become more difficult due to the worldwide increase in multidrug resistant strains, leaving only limited clinical treatment options [4–8]. In effect, about 80% of the nosocomial infections caused by* K. pneumoniae* are due to these multidrug-resistant strains; the incidence of ESBLs (extended-spectrum beta-lactamase) isolates ranges from 8% to 44% [9, 10].

Bacteriophages, or phages, are viruses that specifically attack and kill their host bacteria, a process that is regarded as a possible treatment method for combating bacterial infectious diseases. Bacteriophage treatment is particularly desirable because of the side effects and inefficacy associated with antibiotics and the emergence of new antibiotic-resistant strains. The bacteriophages have garnered increasing attention as an alternative to controlling bacterial infectious diseases with antibiotics since avoiding an antibiotic treatment would avoid the spread of multiresistant bacteria [11–14]. In comparison with antibiotics, phages have many advantageous qualities, such as a high abundance, host specificity, rapid exponential replication, and a decline in numbers when the target bacteria decrease. Furthermore, phage therapy has already been tested in a variety of bacterial species and has accomplished some achievements [15–17]. For example, Chhibber et al. delivered phage via an intraperitoneal route to treat experimental lobar pneumonia that was induced by* K. pneumoniae* in mice and had a great efficacy [9]. However, an intraperitoneal administration of a phage would not be either practical or efficient for treating pneumonia in humans. In the present study, we evaluated the therapeutic efficacy of phage 1513 which was delivered intranasally in order to protect against pneumonia that is induced by a strain of multidrug resistant* K. pneumoniae*.

## 2. Materials and Methods

### 2.1. Bacterial Strain

A* K. pneumoniae* strain was isolated from a patient with pneumonia at the Zhongshan Hospital Affiliated to Dalian University (Dalian, China) and then was designated KP 1513. KP 1513 was identified as a multiple antibiotics-resistant and ESBLs-producing strain by the Walk Away Plus System (Siemen's Diagnostics). Stocks of the strain were stored at −80°C in Luria-Bertani broth (yeast extract 5 g, tryptone 10 g, and NaCl 10 g in 1 L of distilled water) containing 25% glycerol.

### 2.2. Isolation, Enrichment, and Purification of Bacteriophage

KP 1513 was used as a host to isolate a lytic phage from the sewage samples from the Zhongshan Hospital Affiliated to Dalian University. Briefly, 20 mL of the sewage sample was centrifuged at 5000 g for 5 min at 4°C to remove any debris and soil. Then, the supernatant was pooled and supplemented with CaCl_2_ to 1 mmol L^−1^. Ten mL of this supernatant was combined with 10 mL of 2 × LB broth and 2 mL of bacteria solution and was then incubated for 12 h at 37°C with shaking (120 rpm). Then, the enrichment broths were centrifuged at 8000 g at 4°C for 5 min. The supernatant was pooled and passed through a 0.22 *μ*m filter (Millipore, USA). A double-layered agar method was used to confirm phage presence in the filtrate, and the plaque forming unit (PFU) was calculated. Single plaques were subcultured three times for phage purification. The suspension that contained specific phage was incubated with DNase I and RNase (1 *μ*g mL^−1^, Takara, Japan) at 37°C for 30 min prior to adding NaCl (1 mol L^−1^) and polyethylene glycol 8000 (PEG8000; Sigma) to precipitate the phage particles. This phage was designated phage 1513 and was resuspended in SM buffer (NaCl 5.8 g, MgSO_4_·7H_2_O 2 g, Tris·Cl 1 mol L^−1^, and 2% gelatin 5 mL in 1 L of distilled water, pH 7.5). Then, cesium chloride equilibrium ultracentrifugation (150,000 g for 24 h in a Beckman Ti50 rotor) was conducted for further phage purification. The band that contained the phage was collected and dialyzed against a TBS buffer (NaCl 8 g, KCl 0.2 g, and tris base 3 g in 1 L of distilled water, pH 7.4 with pure HCl) at 4°C overnight. Then, phages were passed through a Detoxi-Gel endotoxin removing gel (Pierce Biotechnology, Rockford, IL) to remove endotoxins from the phage preparation. Finally, the purified phages were stored at 4°C until use.

### 2.3. Characterization of Phage 1513

Transmission electron microscopy was used to observe phage 1513 with a JEM-1200EX electron microscope (JEOL, Japan) after negative staining by phosphotungstic acid.

Thermal, pH, and chloroform stability tests were conducted. Aliquots of phage suspensions with high titers (2 × 10^8^ PFU mL^−1^) were incubated at 4°C, 25°C, 37°C, 50°C, 60°C, and 70°C (pH 7) for 60 min or at 4°C (pH 3–12) for 10 h or mixed with chloroform and incubated at 4°C for 24 h. The phage titers were tested by the double-layer agar method.

A one-step growth experiment was carried out by modifying the method described by Ellis and Delbruck [18]. Briefly, the phage was added to broth culture of KP 1513 to a MOI (multiplicity of infection) of 0.1. The sample was then incubated at 37°C for 15 min in order to promote phage-host adsorption. The mixture was centrifuged at 12,000 g at 4°C for 10 min to remove any free phage particles. The supernatant was removed, and the pellet was resuspended in LB broth. The above-mentioned centrifugation procedure was repeated once more. The pellet was resuspended in 100 mL LB medium and incubated at 37°C while shaking. Aliquots were removed at 10 min intervals, and the phage titer was determined by spot method.

Genome sequencing was performed by Shenzhen Genomics Institute (Shenzhen, China) using the Illumina High-Throughput Sequencing Platform (Illumina HiSeq 2000) on DNA obtained by means of standard SDS-proteinase K procedures. The complete genome sequence of the phage 1513 was accessible in GenBank (Accession number KP658157).

### 2.4. Bactericidal Effect of Phage 1513* In Vitro*

An aliquot of overnight culture of KP 1513 was seeded into fresh LB medium and incubated for 6 h at 37°C with shaking (150 rpm). The bacteria were pelleted and resuspended in PBS to an OD_650_ of 0.6 (~2 × 10^9^ CFU mL^−1^). Different phage 1513 concentrations (2 × 10^10^, 2 × 10^9^, and 2 × 10^8^ PFU mL^−1^) or PBS (control) were added to the inocula and then further incubated for 12 h at 37°C with shaking (150 rpm). Bacterial growth was monitored by measuring the OD_600_ at a 30 min interval.

### 2.5. Mice

Seven-week-old female Swiss-Webster mice (weight ~25 g per mouse) were purchased from the Dalian Medical University in Dalian, China (SCXK 2008-0002). All procedures were conducted according to guidelines established by the Ethics Committee of Dalian Medical University.

### 2.6. Mouse Lethal Pneumonia Model

Groups of 10 mice were anesthetized by an IP injection with ketamine (120 mg kg^−1^) and xylazine (12 mg kg^−1^) and inoculated with 20 *μ*L inocula of KP 1513 (2 × 10^8^ CFU/mouse) intranasally. LD_50_ of the KP 1513 was 4.17 × 10^9^ CFU mL^−1^ intranasally in the previous study. In the phage therapeutic experiments, a single dose of phage (2 × 10^9^, 2 × 10^8^, and 2 × 10^7^ PFU/mouse) or PBS was given intranasally 2 h after infection.

### 2.7. Bacterial Burden, Histopathology, and Cytokine Assay of Lung Tissue

Groups of 11 mice were inoculated intranasally with 6 × 10^7^ CFU/mouse of KP 1513 phages (6 × 10^7^ PFU/mouse) or PBS were administered intranasally 2 h after infection. At 24 h after infection, five mice from each group were euthanized, and the left lungs were removed and fixed by 10% formalin for at least 24 h at 4°C and were then sent to the pathology department of the Zhongshan Hospital for the haematoxylin-eosin (HE) stain. Another 6 mice from each group were euthanized and weighed, and their whole lungs were homogenized in sterile saline. An aliquot of the homogenate was frozen at −80°C for cytokine testing (TNF-*α* and IL-6) using the Mouse ELISA Kit (Nanjing Jiancheng, China). The remaining homogenate was diluted and plated onto Luria-Bertani agar in order to determine the colony formation unit. To detect the phage level present in the lung, the homogenate was centrifuged at 4000 g to obtain the supernatants. The supernatants were filtered through a 0.22 *μ*m pore-size filter prior to conducting the double-layered agar method to calculate the number of phages by plaque forming unit (PFU).

### 2.8. Statistical Analysis

A log rank test was performed to test the significant difference between the survival curves. Two-tailed Mann-Whitney paired tests were used to compare weight loss, bacterial quantifications, bacteriophages, and cytokines in the lung sample. Error bars represent s.e.m.

## 3. Results

### 3.1. Isolation and Morphological Study of Phage 1513

**Figure 1:**
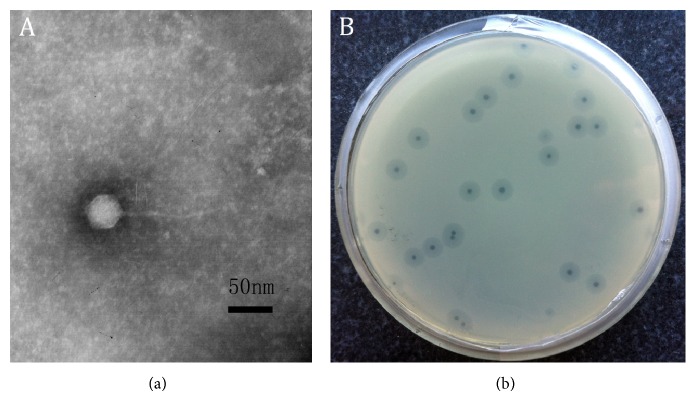
Morphology of* K. pneumoniae* phage 1513. (a) Electron micrograph of phage 1513. (b) Plaque morphologies of phage 1513.

A lytic phage against KP 1513 was isolated from the sewage samples and then purified. By transmission electron microscopy, this phage was identified as a virion particle that was approximately 150 nm long with an icosahedral head (approximately 48 ± 3 nm long) and a tail (approximately 100 ± 10 nm long), which indicates that this phage belongs to the Siphoviridae family (Figure 1). In addition, when cultured with KP 1513, the phage formed clear plaques and was surrounded by a large halo.

### 3.2. Characterization of Phage 1513

**Figure 2:**
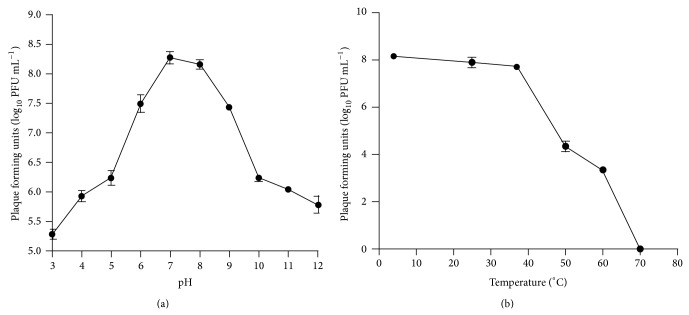
Stability of phage 1513 under different pH (a) or temperature (b).

pH and temperature stabilities are important for the therapeutic application of the phage in animals. In this study, phage 1513 was susceptible with strongly acid and alkaline pH (pH < 3 or pH > 10) and was relatively stable at pH between 6 and 9 at 4°C for 10 h. In addition, phage 1513 was stable at 4°C (for short time storage) and 37°C at pH 7 (condition in lung) (Figure 2). Furthermore, phage 1513 was not chloroform-sensitive, which is important since chloroform was involved in the purification procedures.

**Figure 3:**
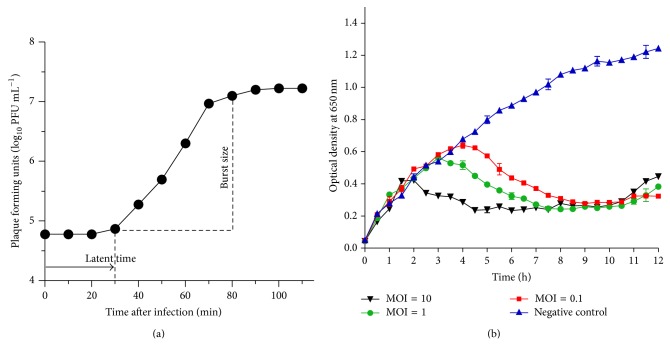
(a) Phage 1513's one-step growth curve was measured. Bacteriophages showed a latent period of about 30 min and a burst size of 264 PFU mL^−1^. (b) Lytic ability of phage 1513* in vitro*. KP (2 × 10^9^ CFU mL^−1^) was infected with the phage at different multiplicities of infection (MOI). The optical density at 650 nm was determined at each timepoint. Infection dose of phage: none (▲), 2 × 10^10^ PFU mL^−1^ (▼), 2 × 10^9^ PFU mL^−1^ (●), and 2 × 10^8^ PFU mL^−1^ (■).

The one-step growth curve revealed that phage 1513 had a latent period of 30 min and an average burst size of 264 (Figure 3). These calculations were based on the ratio of mean yield of phage particles liberated to the mean phage particles that infected the bacterial cells in the latent period, which indicates that phage 1513 can quickly infect the host and propagate.

The complete genome sequence of the phage indicated that the linear double stranded DNA of* Klebsiella* phage 1513 is 49,462 bp in length with 50.61% of G + C, as well as 72 CDSs, which comprised about 82.81% of the genome. Moreover, a tandem repeat sequence (5′-GGTTTCTACGGTTTCGACGGTTTCTAC GGTTTCT-3′, 34 bp) was observed by Tandem Repeats Finder without tRNA gene.

### 3.3. Effect of Phage 1513 against* K. pneumoniae In Vitro*

To assess the ability of the phage to lyse host bacteria* in vitro*, we monitored the host bacteria's growth while being in the phage's presence. After adding phage 1513 to KP 1513 culture, the optical density at 650 nm constantly rose for 2 h, but it then dropped down by different extents according to the MOI (Figure 3). The differences between treatment groups gradually became negligible after 8 h incubation. Since the optical density of the phage-inoculated groups was much lower than that of the control group, we could conclude that phage 1513 was able to effectively lyse KP 1513* in vitro* (Figure 3).

### 3.4. Phage 1513's Effect on Lethal Pneumonia

**Figure 4:**
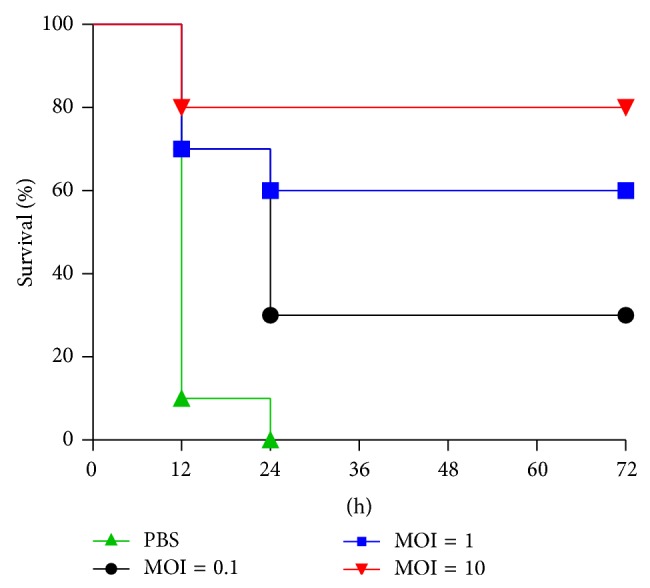
Efficiency of phage 1513* in vivo*. Survival curves of the infected mice treated with either PBS or phages at different multiplicities of infection (MOI). A group of 10 mice were inoculated via the intranasal route with 2 × 10^8^ CFU/mouse* K. pneumoniae*. Various doses of phage 1513 were administrated intranasally 2 h after infection. Survival curves were compared for significance with the log-rank test of the results obtained from the following parameters: from the phage 1513 (2 × 10^7^ PFU/mouse) treatment group versus those from the control group (*P* = 0.0058), from the phage 1513 (2 × 10^8^ PFU/mouse) treatment group versus those from the control group (*P* = 0.0018), and from the phage 1513 (2 × 10^9^ PFU/mouse) treatment group versus those from the control group (*P* = 0.0002).

As shown in Figure 4, without treatment 100% of the mice (10/10) died after an intranasal inoculation of 2 × 10^8^ CFU of KP 1513/mouse. By contrast, all phage 1513 doses that were delivered intranasally 2 h after infection improved the survival in a dose-dependent manner: the highest dose (2 × 10^9^ PFU/mouse) showed the greatest survival rate compared to that of the control group (80% versus 0%; *P* = 0.0002). The survival rate was, respectively, 60% (6/10; *P* = 0.0018) and 30% (3/10; *P* = 0.0058) in the treatment group of MOI = 1 (2 × 10^8^ PFU/mouse) and MOI = 0.1 (2 × 10^7^ PFU/mouse).

### 3.5. Phage Treatment Improved the Lung Damage

**Figure 5:**
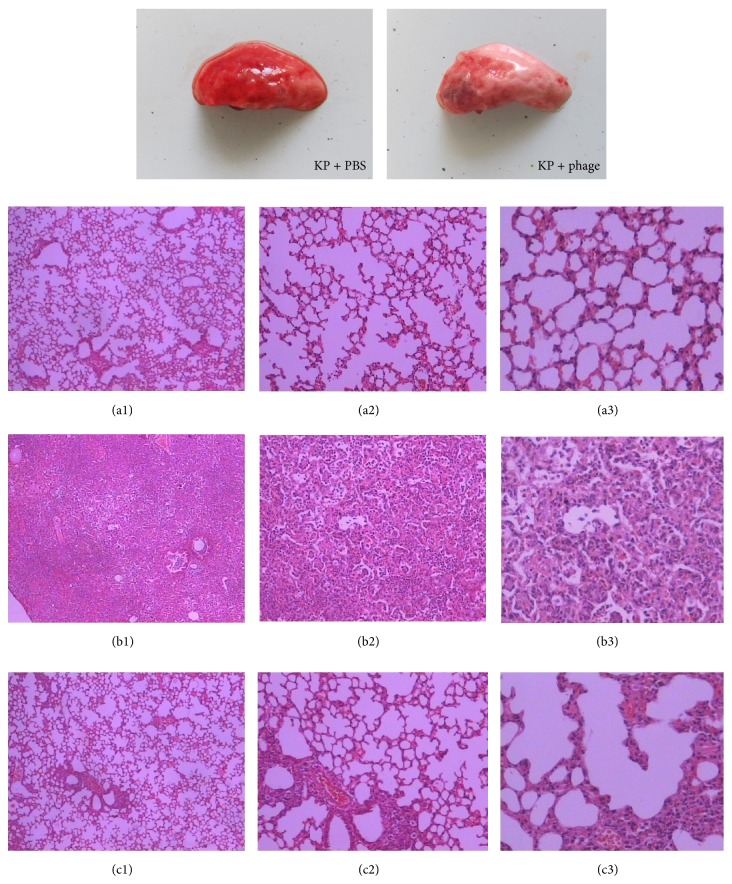
Histopathology of KP-infected lung tissue from PBS- or phage 1513-treated mice. A group of 10 mice were inoculated intranasally with 6 × 10^7^ CFU KP/mouse. Additionally, phage 1513 was administered intranasally. The mice were euthanized 24 h after infection, and lung tissues were removed as described above. (a) PBS, without* K. pneumoniae*; (b)* K. pneumoniae*, treated with PBS; (c)* K. pneumoniae*, treated with phage 1513 ((a1), (b1), and (c1) were amplified 40 times; (a2), (b2), and (c2) were amplified 100 times; and (a3), (b3), and (c3) were amplified 200 times).

The pathology of the infected lung tissues showed focal consolidation areas in the phage-treated mice, whereas diffuse consolidation was observed in the PBS-treated mice. Furthermore, the results of histopathologic sections of the infected lung tissues were analogous. In the control group, the pulmonary alveoli exhibited an abnormal structure, and the majority of the alveolar space was obliterated by inflammatory exudate and immune cell infiltrate, which was accompanied with hemorrhage and consolidation. In contrast, the phage-treated mice's lungs showed local, discrete lesions, and most of the tissue was healthy (Figure 5).

### 3.6. Phage Treatment's Effect on Body Weight Loss, Bacterial Burden, and Cytokines Released in Lung

**Figure 6:**
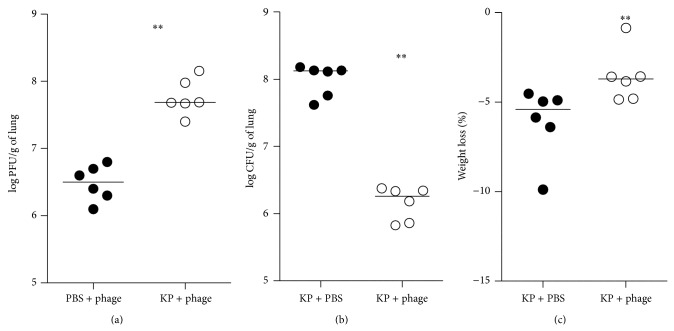
Body weight loss, bacterial burden, and the phage number present in the lung after phage treatment of* K. pneumoniae* infection. Groups of 6 mice were inoculated via the intranasal route with 6 × 10^7^ CFU/mouse* K. pneumoniae*. PBS or phage 1513 (6 × 10^7^ PFU/mouse) was administered intranasally 2 h after infection. The numbers of bacteriophage (a) or bacteria (b) in the lung homogenates were determined. Mice were weighed (c). Bacteria counts and weight loss rate were significantly lower in the lung homogenate from the mice in the phage-treated group than in the control group (*P* = 0.0022 and *P* = 0.0087). Bacteriophage counts were significantly higher in the infected group than in the noninfected animals (*P* = 0.0022).

**Figure 7:**
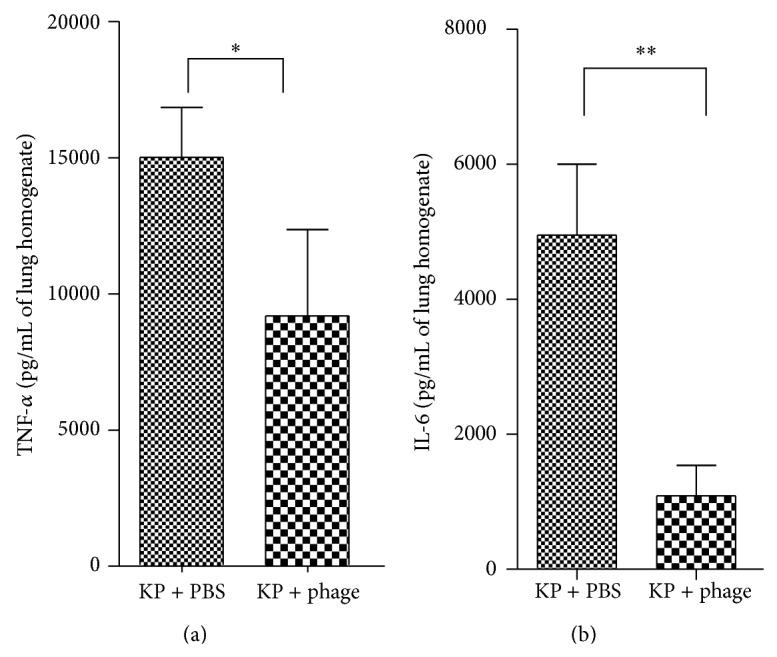
Cytokine assay of lung tissue after phage treatment of* K. pneumoniae* infection. Groups of 6 mice were inoculated via the intranasal route with 6 × 10^7^ CFU/mouse* K. pneumoniae*. PBS or phage 1513 (6 × 10^7^ PFU/mouse) was administered intranasally 2 h after infection. The mice were killed 24 h after infection, and the inflammatory cytokines of the lung homogenate were collected and detected. Cytokine concentrations were remarkably lower in the phage-treated group than in the control group. (a) TNF-*α* (*P* = 0.0159). (b) IL-6 (*P* = 0.0079).

In the phage-treated group, the mice's weight loss rate (weight difference before infection and 24 h after infection/weight before infection) was significantly lower. In the homogenate of lungs, the number of bacteria (log^10^ CFU g^−1^) in phage group and control group was, respectively, 6.16 ± 0.10 and 7.99 ± 0.10, so the number of bacteria was approximately two log units lower than that in the control group. The number of bacteriophage (log^10^ PFU g^−1^) in phage group and control group was, respectively, 6.48 ± 0.11 and 7.76 ± 0.11, so the number of phage was more than 10 times higher in the treatment group (Figure 6). In addition, cytokine levels in the phage-treated mice were remarkably lower (TNF-*α*, *P* = 0.0159; IL-6, *P* = 0.0079) than those of the control mice (Figure 7).

## 4. Discussion

Hospital-acquired pneumonia that is caused by* Klebsiella pneumoniae* is always a threat as well as a fastidious public, human health problem [19]. Despite advances in antimicrobial therapy, the morbidity and mortality remain high and out of control. Furthermore, the emergence of multidrug resistance aggravates this situation. An increasing number of extended-spectrum-*β*-lactamase-producing and KPC-type carbapenemases-producing* K. pneumoniae* nosocomial isolates have been reported [4, 7, 20–22]. Since antibiotic treatment has associated restrictions and shortcomings, phage therapy is now more frequently being considered as a potential treatment and prevention for bacterial infections [23]. In the present study, we isolated a new bacteriophage (phage 1513) that could effectively control pneumonia that is caused by a clinical multidrug resistance* K. pneumoniae in vitro* and* in vivo*.

The* in vitro* study showed phage 1513's effectiveness against* K. pneumoniae* with a short latent time and a large burst size. In addition, the phage possessed stability within physiological ranges of temperature and pH. It formed plagues on 5 of 10 clinical* Klebsiella* strains tested, notably KP 1513. Plaques did not exist on* Pseudomonas aeruginosa* (ATCC27853),* Staphylococcus aureus* (Newman), and* E. coli* (CGMCC 1.797). It was consistent with previous documents that bacteriophage are highly specific and can only infect a single species of bacterium, usually a subset of strains within that species [13, 24]. Therefore, phage 1513 has a narrow spectrum and is potentially active against the MRKP strains.

However, it can be found that an early trend of phage resistance emerged after 11 hours of incubation (Figure 3). Mutants that are resistant to phage infections are a critical problem for the application of phage therapy [25]. However, some studies have demonstrated that the resistance is partially due to receptor molecule variation, which acts as virulence factor of pathogens. As a result, bacteria have to attenuate their virulence in order to be resistant to the phage lysis [26]. On the other hand, a phage can also mutate to adapt to the change of pathogens [27]. Further investigations are required to determine whether the phage-resistant* K. pneumoniae* cells that have emerged through phage therapy have reduced the pathogenic ability. Perhaps a cocktail of several phage strains will be necessary for controlling bacterial variation.

Chhibber et al. have demonstrated that a single intraperitoneal administration of a high bacteriophage dose, which was 100 times the infectious bacterial dose, administered immediately after the intranasal infection has been shown to rescue 100% of animals [9]. As we know, the delivery route has a critical influence on phage therapy. People rarely deliver intraperitoneally in clinical treatments; however, they take drugs by nebulisation, which is similar to intranasal administration. Compared with intranasal injection, intraperitoneally administering the phage is more suitable for systemic infections but not for local infections, like pneumonia. Moreover, if phages were delivered intraperitoneally, they would be detected and cleared out more quickly by the immune system. In this study, we demonstrated that intranasal administration can also effectively treat pneumonia that is caused by* K. pneumoniae* in mice. We gave a various dose of phage (2 × 10^9^ PFU/mouse, 2 × 10^8^ PFU/mouse, 2 × 10^7^ PFU/mouse) 2 h after infection and, as a result, the survival rate was affected. This is probably because the increasing dose of bacteriophages results in more rapid bacterial killing, thereby increasing the survival rate [28]. Besides, phage 1513 cannot provide significant protection 24 h prior to infection (data not shown). It is speculated that the phage has been eliminated before it attached to the host and cracked it. The clearance rate of the phage particles from body fluids by the reticuloendothelial system is a critical parameter for phage therapy [26]. It may be more effective to increase the dose of phage or give it less than 24 h before infection. But phage 1513 has obvious advantages on account of the significant effect in the treatment experiment.

In addition, from Figure 6, we can see that bacteria numbers significantly decreased in the phage-treated mice, which suggests that the phage effectively killed* K. pneumoniae in vivo*. Consequently, in the phage-treated mice, lung lesions and an inflammatory response were clearly less prominent in comparison to the control. These were all concordant with the survival study results. Moreover, many reports have indicated that a cytokine storm results in lung injury and poor clinical outcomes [29, 30]. Therefore, the phage may be beneficial to the lung by decreasing the host's inflammatory response, as demonstrated by the levels of IL-6 and TNF-*α*.

In conclusion, the results of this study suggest that a phage treatment that is administered intranasally has great potential for treating pneumonia and other infections caused by* K. pneumoniae*. The safety of phage 1513 and its activity against biofilm formation will be investigated in further study.
